# Adaptation of Muscles With Different Physiological Properties to Resistance Training With and Without Bloodflow Restriction

**DOI:** 10.1177/19417381261459244

**Published:** 2026-07-03

**Authors:** Margarida Cidrais, Carolina Teodósio, Joana M. Correia, Carolina Vila-Chã, Pedro Pezarat-Correia, Paula M. Bruno, Goncalo V. Mendonca

**Affiliations:** † Neuromuscular Research Lab, Faculdade de Motricidade Humana, Universidade de Lisboa, Estrada da Costa, Cruz Quebrada, Dafundo, Portugal; ‡ Higher School of Sport, Wellbeing and Biomedical Systems, Polytechnic University of Cávado and Ave, Barcelos, Portugal; § Egas Moniz School of Health and Science, Caparica, Monte de Caparica, Almada, Portugal; ‖ CIPER, Faculdade de Motricidade Humana, Universidade de Lisboa, Estrada da Costa, Cruz Quebrada, Dafundo, Portugal

**Keywords:** exercise physiology, muscle physiology, strength and conditioning, ultrasound

## Abstract

**Background:**

Low-load bloodflow-restricted (BFR) resistance training (LLBFR-RT) has been shown to elicit muscular hypertrophy comparable with high-load resistance training (HL-RT). We aimed to explore the differential impact of LLBFR-RT and HL-RT on hypertrophy of the triceps surae.

**Hypothesis:**

LLBFR-RT would be more effective than HL-RT for soleus muscle hypertrophy. In addition, it was hypothesized that HL-RT would be more effective than LLBFR-RT for the hypertrophy of both gastrocnemii.

**Study Design:**

An active controlled trial.

**Level of Evidence:**

Level 2.

**Methods:**

A total of 28 healthy adults were assigned randomly to 2 unilateral plantar-flexion training regimens: HL-RT (75% of 1-repetition maximum [1RM], 4 sets, 10 repetitions) and LLBFR-RT (20% of 1RM, 4 sets, 30+15+15+15 repetitions). Maximal voluntary isometric contractions (MVIC), 1RM, and panoramic ultrasound assessments of the triceps surae were obtained pre- as well and post-4 weeks of intervention (5 sessions per week).

**Results:**

MVIC and 1RM improved statistically from pre- to post-training (*P* < 0.05), and this occurred similarly between interventions (gains of ~20% to 30%). Both regimens were similarly effective for the hypertrophy of the soleus muscle (gains of ~12% to 15%). Conversely, the lateral gastrocnemius only responded to HL-RT (gains of ~12%; *P* < 0.05 vs 4% with LLBFR-RT), and the medial gastrocnemius did not manifest statistical changes with either training regimen.

**Conclusion:**

HL-RT is more effective for eliciting size gains in the lateral gastrocnemius (muscle with a mixed fiber type composition) over a period of high-frequency training. The soleus (muscle with predominant oxidative fibers) adapts to both training regimens in a similar fashion.

**Clinical Relevance:**

High-frequency plantar-flexion exercise with HL-RT (5 days per week) is preferable to LLBFR-RT when aiming to elicit plantar-flexor strength gains accompanied by more comprehensive hypertrophy of the triceps surae muscle.

Resistance training has a beneficial impact on health and sports performance.^16,50^ However, some people (e.g., beginners, older adults, people with chronic conditions or those recovering from injuries) may be unable to perform or tolerate heavy-load resistance training (HL-RT, typically set at >60% 1-repetition maximum [1RM]).^44^ In those circumstances, alternative methods—namely, low-load blood-flow restricted (BFR) resistance training (LLBFR-RT)—can offer a safer and still-effective alternative for improving muscle strength and promoting hypertrophy.^5,31^ Notably, LLBFR-RT has been shown to elicit muscular hypertrophy comparable with that elicited by HL-RT.^31^ Combining both LLBFR-RT and HL-RT has also been shown to enhance motor performance in healthy athletes.^51^ There is some evidence that full-body LLBFR-RT can increase lean body mass, muscle strength, power, and endurance in young physically inactive men and women.^38^ Therefore, LLBFR-RT can be used for eliciting muscular adaptations in a broad spectrum of people to whom strength-based interventions can be particularly relevant.^5,31,38,51^

Although most studies have shown that the magnitude and timeline of whole muscle growth is similar between LLBFR-RT and HL-RT,^5^ recent meta-analytic findings indicate that type I muscle fiber hypertrophy is at least as great as, and sometimes greater than, type II hypertrophy when performing LLBFR-RT >5 days per week.^45^ This is in contrast to the predominant hypertrophy of type II muscle fibers typically observed after HL-RT.^17^

Fiber type composition varies between muscles and the differences in fiber type composition are related to differences in the functional characteristics of the muscle. For instance, while postural muscles exhibit a higher proportion of type I muscle fibers, this is not the case for phasic muscles.^9^ The triceps surae is a 3-headed muscle (soleus, lateral, and medial gastrocnemius) that represents a convenient model to explore the impact of different training regimens on muscles with different proportions of type I and type II fibers.^46^ For instance, while the soleus contains predominantly type I muscle fibers (~80%), the proportion of type I fibers in the gastrocnemii is ~57%.^19^ Thus, plantar-flexion training regimens might have the potential to induce greater or smaller hypertrophy on the soleus or the gastrocnemii depending on the loading scheme being implemented over time.^15^ Hypothetically, schemes involving lower loads and a higher number of repetitions would target primarily the soleus and those with higher loads and fewer repetitions would target primarily the gastrocnemii muscles. However, previous findings cast doubt on the claim that training muscles based on their fiber composition provides any benefit for enhancing muscle strength or hypertrophy.^46^ Despite this, it is conceivable that some properties unique to LLBFR-RT (e.g., greater ischemia, reperfusion, and metabolite accumulation) may enhance the stress on type I fibers in response to training.^45^ Indeed, if LLBFR-RT represents an effective means of stimulating the hypertrophy of type I muscle fibers, post-training adaptations in muscle cross-sectional area (CSA) would be expected to become more evident in the soleus. Conversely, if type II hypertrophy is substantially greater than that of type I myofibers with HL-RT,^17^ the gastrocnemii might respond more profoundly to this loading scheme. To our knowledge, only 3 previous studies have examined the differential impact of LLBFR-RT versus HL-RT on plantar-flexor muscle hypertrophy.^3,24,35^ Unfortunately, in 2 of these studies, chronic adaptations were reported only for the medial gastrocnemius or the soleus.^3,35^ In 1 study, the authors obtained muscle thickness of both the gastrocnemius and the soleus. However, the design of this study involved training one leg with LLBFR-RT and the other with HL-RT in the same exercise session.^24^ This represents a limitation because BFR applied to one limb may enhance indirectly the training effects of other exercised muscles that are not under BFR.^32^ Therefore, based on the available research, it is not possible to draw more definite conclusions on this topic. For this reason, the present study sought to explore whether the 3 heads of the triceps surae manifest a differential hypertrophic response to LLBFR-RT versus HL-RT. It was hypothesized that LLBFR-RT would be more effective than HL-RT for soleus muscle hypertrophy. In addition, it was hypothesized that HL-RT would be more effective than LLBFR-RT for the hypertrophy of both gastrocnemii.

## Methods

### Study Design

This study employed a pre-post design whereby participants were assigned randomly (via a computer-generated sequence) to 2 unilateral plantar-flexion training regimens: (1) LLBFR-RT at 20% of 1RM; or (2) HL-RT at 75% 1RM. All experimental procedures were conducted in the morning between 07:00 and 11:00 in a temperature-controlled laboratory (21°C to 23°C). Participants were familiarized with the experimental protocol 1 week before testing. Familiarization consisted of 1 session designed to ensure the adaptation to all testing procedures and to test whether participants were able to perform at least 25 standing heel-raises with their dominant limb. Then, each participant completed 20 unilateral training sessions over 4 weeks (5 sessions per week). The intervention protocol allowed each participant to complete a sufficient training volume to ensure comparable adaptations in muscle strength between LLBFR-RT and HL-RT (>18 sessions).^5^ Assessments of 1RM and maximal voluntary isometric contraction (MVIC) were obtained at pre- and post-training timepoints. Panoramic ultrasound assessments were used to determine changes in the anatomical CSA of the soleus, medial, and lateral gastrocnemius muscles from pre- to post-training. All post-training measurements were obtained 48 hours to 72 hours after the last training session.

### Participants

Based on past research, the partial eta-squared values for the effects of LLBFR-RT and HL-RT on MVIC and soleus hypertrophy corresponds to 0.23 and 0.19, respectively.^35^ Considering an alpha level of 0.05, a sample of 28 participants (n = 14 per group) was estimated to achieve 80% of power of correctly rejecting the null hypothesis (2 measurements, 2 groups). In addition, assumptions of nonsphericity correction factors were incorporated into the power analysis to ensure appropriate adjustments to the sample size calculation (G*power software, Version 3.1.9.7). The recruitment was expanded to 40 participants to accommodate an attrition rate of 30%.

Young healthy volunteers (20 men, 20 women) were recruited from the local communities. Participants were free from any use of medication and exhibited values within the healthy range for body-mass index (18.5 kg/m^2^ to 24.5 kg/m^2^) and blood pressure (systolic and diastolic blood pressure <120 mmHg and <80 mmHg, respectively). Before enrollment, all participants completed a health screening questionnaire. Exclusion criteria included current smoking habits, a history of metabolic, cardiovascular, or respiratory conditions, and any musculoskeletal limitations that could impair exercise performance. Volunteers who had participated in regular RT (frequency ≥2 exercise sessions per week) within the previous 8 weeks were not included. This is important because past research has shown that the individual training status moderates the effects of LLBFR-RT on gains of muscle strength and size.^18^ Moreover, all potential participants were required to complete a questionnaire to determine the degree of footedness.^13^ Only those with a score ≥20 (strong lateral dominance) were enrolled in this study.^14^ In addition, inclusion was limited to participants that were able to perform a minimum of 25 standing heel-raises with the dominant limb at study entry.

The effect of the menstrual cycle on the dependent variables was not controlled in this study. All female participants reported having spontaneous and regular menstrual cycles ranging from 24 days to 35 days over the preceding 3 cycles. None had used hormonal contraceptives for at least 6 months before the study. Testing sessions were conducted approximately 2 hours after the participants’ last meal. In addition, participants were instructed to refrain from any form of exercise for at least 24 hours and to avoid caffeine consumption on testing days. This study complied with the principles set forth in the Declaration of Helsinki and its latter amendments or comparable ethical standards. The experimental design was approved by the Faculty’s Ethics Committee (CEFMH no. 4/2017). The risks of participation were explained carefully and informed consent was obtained from all participants.

### Maximal Voluntary Isometric Contraction

Testing was conducted unilaterally on a Biodex System 3 Pro isokinetic dynamometer (Biodex Medical Systems), with the participants’ hips and knee flexed at 120º (full knee and hip extension equivalent to 180º) and their ankle positioned at 110º of plantar flexion. All participants completed a general warm-up consisting of 3 to 4 isometric subjectively graded submaximal isometric contractions. After 5 minutes of recovery, MVIC was determined for plantar flexion based on 3 isometric contractions lasting 5 seconds each. Participants were instructed to exert their maximum force and subsequently were allowed to rest for 60 seconds between trials. Online visual feedback of the torque exerted was displayed on a computer monitor. The highest peak torque value obtained during plantar flexion was recorded as MVIC. The dynamometer torque signal was sampled at a 1000 Hz analog-to-digital conversion rate by using an external analog-to-digital converter (National Instruments, USB-6251). The signal was then smoothed offline using a digital fourth-order, zero lag Butterworth filter with a cutoff frequency of 10 Hz. Based on previous research from our laboratory, the intraclass correlation coefficient and the 1.96 × standard error of the measurement for plantar-flexion peak torque using this methodology corresponds to 0.99 and 2.86 Nm.^34^

### One-Repetition Maximum

Unilateral plantar-flexion 1RM of the participants’ dominant right leg was assessed before and after training. The individual 1RM was also determined whenever the participants were able to complete ≥2 additional repetitions over their assigned goal for the last set in 2 consecutive training sessions. The purposes of the additional assessments of 1RM were to: (1) enable the adjustment of absolute training loads to 20 (LLBFR-RT) and 75% (HL-RT) of 1RM when necessary, and (2) minimize the influence of the strength tests themselves, which inherently favor the performance of participants allocated to the HL-RT group as external loads used in training are much greater than those of LLBFR-RT. This is relevant because past research has shown that the opportunity to perform several strength assessments over time may attenuate these differences in strength.^47^

Participants’ 1RM was tested on the same calf rotary machine used for training (Technogym, Gambetolla). The assessment began with a set of 8 to 10 repetitions at approximately 50% of the estimated 1RM, followed by a set of 5 repetitions at 75% of the estimated 1RM. These initial sets served as a specific warm-up. Afterward, the load was adjusted accordingly and the participants attempted to perform >2 complete repetitions with verbal encouragement. If >2 repetitions were completed before failure, the load was increased by 5%. A rest period of 3 minutes was provided between the first 3 attempts, with 5 minutes of rest allowed for any subsequent trials to ensure full recovery. A repetition was considered valid only if the participant completed the full range of motion (i.e., from full dorsiflexion to maximum plantar flexion). Participants were instructed to keep their knee fully extended and their foot pointing forward during all testing sessions; 1RM was accepted as the maximum load that each participant could mobilize in a single maximum dynamic plantar flexion

### Panoramic Ultrasound Assessment

All images were taken at 30% of the proximal lower-limb length (distance from the articular cleft between the femur and tibia condyles to the lateral malleolus). Site markings were made on the skin with indelible ink while the participants remained seated with the right leg relaxed and free from support. Skin markings were remarked with ink at the beginning of each training session to ensure that all pre- and post-training measurements were taken at the same anatomical site. Transverse ultrasound images of the soleus, medial, and lateral gastrocnemius muscles were obtained at all timepoints with participants lying prone on a padded table, with their legs fully extended, completely relaxed, and feet hanging over the edge of the table.^41^

Data were collected using a B-mode ultrasound imaging device (EUB-7500; Hitachi Medical Corporation, Chiyoda-ku) with a 6-cm, 10 MHz linear probe, always operated by the same technician. The scanning head was placed perpendicular to the underlying tissues and was coated with water-soluble transmission gel, which provided acoustic contact without depressing the dermal surface. The probe was then moved manually with slow and continuous movement to acquire images of the soleus, medial, and lateral gastrocnemius. When the quality of the image was deemed to be satisfactory, the technician saved the image to a hard drive for later analysis. This was performed as many times as necessary until 3 good-quality images for each muscle were collected. The outline of the anatomical CSA area of each muscle was first identified by the examiner (always the same technician) and then performed by a tool of ImageJ software (version 1.54; National Institutes of Health) by manual tracking.^12^ The anatomical CSA represents the mean of 3 measurements obtained for each tracked muscle.

Based on test-retest measurements involving 10 participants (not included in this study), the reliability of CSA was compatible with a coefficient of variation (CV) of 4.6% and an intraclass correlation coefficient (ICC) of 0.98 for the soleus muscle. The CV and ICC obtained for the medial gastrocnemius corresponded to 1.6% and 0.99, respectively. Finally, for the lateral gastrocnemius there was a CV of 2.8% and an ICC of 0.97. For these analyses, the same technician captured multiple images from each of the 10 participants and then analyzed each of the images for each participant.

### Resting Arterial Occlusion Pressure

All measurements were taken at the beginning of each BFR training session, while the participants remained seated at rest. During the measurement of arterial occlusion pressure at the beginning of each training session, blood flow was detected using a vascular Doppler probe placed over the right posterior tibial artery, just behind the medial malleolus (SONOLINE B LCD Fetal Doppler 8 MHz vascular probe, CONTEC). Pulse detection was confirmed via auditory and visual feedback from the Doppler device. A 13 × 124 cm pneumatic cuff (SC12L Tourniquet Cuffs, D.E. Hokanson, Inc) was applied to the most proximal portion of the right thigh and inflated using a rapid inflation system (E20 Rapid Cuff Inflator, D.E. Hokanson, Inc). The cuff was first inflated to 50% of the participant’s resting systolic blood pressure and then gradually increased until the tibial pulse disappeared. Arterial occlusion pressure was recorded as the closest 1 mmHg value at which the pulse was no longer detectable.

### Training Regimens

Absolute load was adjusted progressively based on 1RM values obtained individually on a frequent basis (set at 75% and 20% 1RM for HL-RT and LLBFR-RT, respectively). All BFR sessions were completed at 60% of arterial occlusion pressure taken at the beginning of each session. HL-RT was prescribed for 4 sets of 10 repetitions (2 seconds eccentric; 0 seconds pause; 2 seconds concentric; 0 seconds pause), with an interset passive recovery time of 60 seconds.^42^ In both groups, participants completed the plantar flexion exercise through the entire range of motion (calf-rotary machine) while maintaining full knee extension and the foot pointing forwards (neutral position). All participants trained unilaterally, with their dominant (right) leg. LLBFR-RT training involved 4 sets of 30+15+15+15 repetitions (1 second eccentric; 0 seconds pause/1 second concentric; 0 second pause). The recovery time between sets was 30 seconds of passive rest with sustained BFR. This exercise protocol has been used widely in studies examining the physiological responses to BFR resistance exercise (e.g., Loenneke et al^28^). BFR was elicited by inflating a nylon cuff (the same exact cuff that was used for measuring arterial occlusion pressure) in the most proximal portion of the thigh. Before exercise, cuff pressure was adjusted progressively to the intended BFR value (total BFR time approached 275 seconds per session, including exercise and interset recovery periods). All repetitions were guided metronomically to ensure that the participants respected the prescribed movement tempo. Finally, cuff pressure was released immediately after the completion of the last set of LLBFR-RT.

### Statistical Analysis

The normality and equality of variances of the data were tested with the Shapiro-Wilk and Levene’s test, respectively. Baseline group differences in dependent variables were explored with independent samples *t* tests. Two-way mixed-factorial analyses of variance (group [2]: LLBFR-RT vs HL-RT × time [2]: pre- vs post-training) were used to determine the effects of each training regimen on muscle strength and hypertrophy. When a significant effect was detected at a significance level of *P* < 0.05, paired samples *t* tests were used for post hoc comparisons. Adjustment for multiple comparisons was made with Bonferroni’s correction. The partial eta-squared values are reported to indicate effect sizes (ESs) for significant findings (corresponding to a small, medium, and large ESs of 0.01, 0.06, and 0.14, respectively).^7^ Finally, independent samples *t* tests were computed to compare the percent change in muscle strength and hypertrophy resulting from each training regimen. All data are reported as means and standard deviations. Statistical analyses were performed using SPSS Version 27.0 (SPSS, Inc.) and statistical significance was set a priori at *P* < 0.05.

## Results

A total of 28 young healthy volunteers (15 men, 13 women), divided into 2 groups of 14 participants (LLBFR-RT and HL-RT), completed the study protocol. A total of 12 participants were excluded (5 men and 7 women) due to one of the following reasons: history of high-blood lipid levels (2 women, 1 man), history of high blood-glucose levels (1 woman), performance lower than 25 standing heel-raises at baseline (1 woman, 1 man), adherence to each training regimen <100% (3 women, 3 men). All participants whose data was considered for analysis exhibited a 100% rate of adherence to training both regimens during the course of the study.

**Figure 1.**
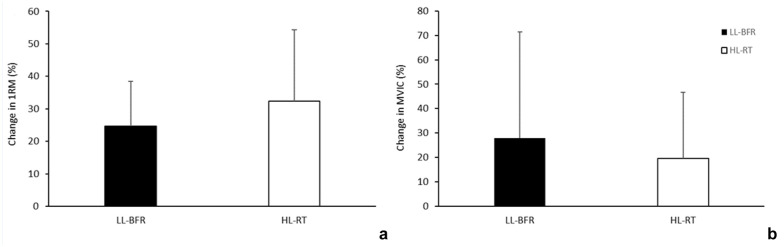
Percent changes in (a) 1RM and (b) MVIC after 4 weeks of LLBFR-RT and HL-RT. 1RM, 1-repetition maximum; HL-RT, high-load resistance training; LLBFR-RT, low-load blood-flow restricted resistance training; MVIC, maximum voluntary isometric contraction.

**Figure 2.**
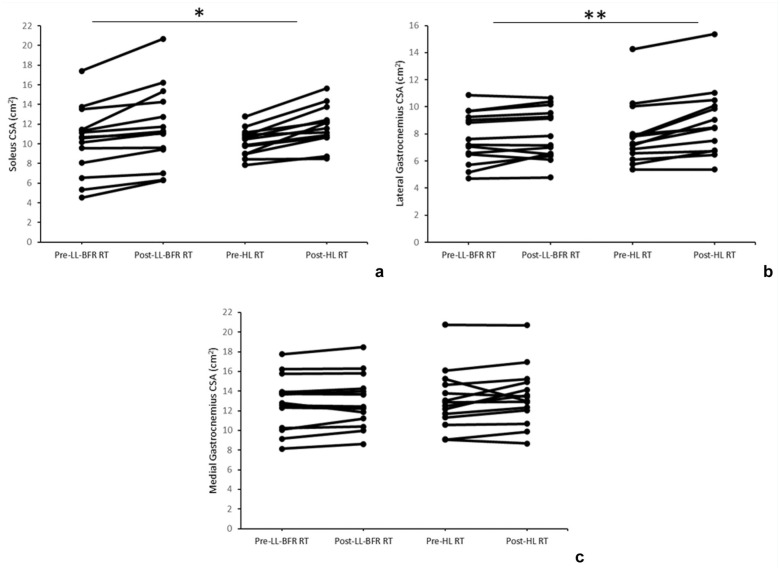
CSA of the (a) soleus, (b) lateral, and (c) medial gastrocnemius before and after 4 weeks of LLBFR RT and HL RT. *P < 0.001 (main effect of time); **P = 0.007 (group-by-time interaction). 1RM, 1-repetition maximum; CSA, cross-sectional area; HL-RT, high-load resistance training; LLBFR-RT, low-load blood-flow restricted resistance training; MVIC, maximum voluntary isometric contraction.

As can be seen in Table 1, at study entry there were no between-group differences in age, height, body mass, or body mass index. Muscle strength and CSA were also similar between groups at baseline (Table 2). The participants’ plantar-flexion muscle strength increased after training in both groups (1RM: only main effect of time: *F* = 57.8, *P* < 0.001; ES, 0.69; MVIC: only main effect of time: *F* = 14.5, *P* < 0.001; ES, 0.36) (Table 2). The percent change in 1RM and MVIC over time was not statistically different between groups (Figure 1a,b). Figure 2 shows the individual CSA responses of the soleus, lateral, and medial gastrocnemius to LLBFR-RT and HL-RT. Both interventions were effective for increasing the CSA of the soleus muscle (only main effect of time: *F* = 40.8, *P* < 0.001; ES: 0.61) (Table 2). As with muscle strength, there were no differences between groups for the percent increase in the soleus CSA between time points (LLBFR-RT: 12.0 ± 12.9%, HL-RT: 15.5 ± 9.5%; *t* = –0.8, *P* = 0.42). Conversely, HL-RT resulted in a statistically significant increase in the CSA of the lateral gastrocnemius, but this did not occur with LLBFR-RT (group-by-time interaction: *F* = 8.7, *P* = 0.007; ES: 0.25) (Table 2). The percent change in CSA of the lateral gastrocnemius was significantly greater (*t* = –2.4, *P* = 0.02) with HL-RT (12.0% ± 9.5%) than with LLBFR-RT (3.9% ± 8.3%). Contrasting with these findings, there were no statistical effects of either training intervention on the CSA of the medial gastrocnemius (Table 2 and Figure 2c).

**Table 1.** Characteristic parameters obtained before 4 weeks of LLBFR-RT and HL-RT

	LLBFR-RT (7 men, 7 women)	HL-RT (8 men, 6 women)	*P* value
Age, years	22.4 ± 3.0	22.0 ± 3.4	0.77
Height, cm	169.9 ± 0.09	167.3 ± 0.08	0.41
Body mass, kg	64.5 ± 9.8	62.5 ± 9.4	0.58
Body mass index, kg/m^2^	22.3 ± 2.4	22.2 ± 2.2	0.96

Values are mean ± SD. HL-RT, high-load resistance training; LLBFR-RT, low-load blood-flow restricted resistance training.

**Table 2:** Plantar-flexion 1RM, MVIC, and CSA of the triceps surae muscle obtained in the trained leg of each group at different time points

	LLBFR-RT		HL-RT	
Variable	Pre-training	Post-training	Pre-training	Post-training
Plantar-flexion 1RM, kg*^a^*	51.7 ± 10.4	64.3 ± 13.7	49.1 ± 7.6	64.8 ± 14.5
Plantar-flexion MVIC, N.m*^a^*	52.7 ± 19.7	61.6 ± 16.1	51.7 ± 14.6	60.9 ± 18.3
CSA medial gastrocnemius, cm^2^	12.9 ± 2.8	13.1 ± 2.7	13.0 ± 3.0	13.3 ± 3.1
CSA lateral gastrocnemius, cm^2 *b*^	7.7 ± 1.9	8.0 ± 1.9	7.9 ± 2.3	8.9 ± 2.5
CSA soleus, cm^2 *a*^	10.4 ± 3.2	11.7 ± 4.0	9.8 ± 1.9	11.4 ± 2.4

Values are mean ± SD. 1RM, 1-repetition maximum; CSA, cross-sectional area; HL-RT, high-load resistance training; LLBFR-RT, low-load blood-flow restricted resistance training; MVIC, maximum voluntary isometric contraction.

a Time main effect (*P* < 0.001).

b Interaction main effect (*P* < 0.01).

## Discussion

To our knowledge, this is the first study to compare the hypertrophic responses to LLBFR-RT and HL-RT in muscle with a mixed-fiber type composition (medial and lateral gastrocnemius) and in muscle with a predominantly slow-twitch composition (soleus). Our primary finding was that both training regimens elicited a similar magnitude of hypertrophy in the soleus muscle. Conversely, while HL-RT was effective for the hypertrophy of the lateral gastrocnemius, this was not the case for LLBFR-RT. Despite this, the gains in muscle strength resulting from training did not differ between both modalities.

### Muscle Hypertrophy

Fisher et al^15^ suggested that superior muscular adaptations can be obtained by training muscles predominant in type I fibers with lighter loads and those predominant in type II fibers with heavier loads. While some human studies corroborate this notion of specific fiber-type hypertrophic effects,^39,49^ others do not.^27,36,46^ Such conflicting results might arise from the fact that those showing differences in the adaptation of muscle fibers did not train to volitional failure, while those showing no differences did. However, neither of these past reports can be compared directly with the present study. First and foremost, LL-RT (prescribed in all previous studies) does not necessarily result in similar adaptations as LLBFR-RT, especially when performed to a predetermined number of repetitions. For instance, under these circumstances, there is enough meta-analytic data to suggest that LLBFR-RT is superior to non-BFR LL-RT in terms of gains in muscle strength and hypertrophy.^30^ Second, the contamination of adaptation between limbs resulting from exercising both legs with different loads in a single training session cannot be totally excluded in 1 of these studies.^46^ In this context, muscle-strength adaptations can become biased due to the contralateral gains resulting from the cross-education effect.^37^ In addition, there are postexercise changes in systemic mediators (e.g., testosterone and growth-hormone) that might also interact with previously exercised muscle from the contralateral limb.^32,33^ This does not mean that a between-subjects design (as used in the present study) is entirely free of limitations. For instance, there could be differences in muscle architecture, neural innervation and activation, and blood supply that are accounted for in a within-subjects design, but not in a between-subjects design.

One previous study, designed specifically to compare the impact of LLBFR-RT versus HL-RT on plantar-flexion muscle strength and triceps surae hypertrophy (in well-trained people training 3 times a week for 6 weeks), observed no effects of either intervention.^24^ Again, these findings cannot be compared with those obtained here because this experimental design also compared legs (assigned to different RT approaches), instead of individual participants. This is important because there is some evidence that BFR applied to one limb may indirectly enhance the size of other exercised muscles.^32^ In the present study, we found that each training regimen exerted a differential effect on the hypertrophy of the triceps surae muscle. Specifically, while the hypertrophic effects of LLBFR-RT were limited to the soleus, this was not the case for HL-RT which also increased the CSA of the lateral gastrocnemius (by ~12%). Therefore, there was a greater partitioning hypertrophy of the triceps surae with LLBFR-RT with emphasis on the soleus (a muscle composed predominantly of type I fibers). Despite this, it cannot be said that LLBFR-RT was more effective than HL-RT for stimulating the hypertrophy of the soleus. Essentially, both interventions increased the CSA of the soleus muscle by around 12% to 15% after just 4 weeks of training. Thus, LLBFR-RT does not seem to be superior to HL-RT for the specific purpose of stimulating hypertrophy of the soleus muscle. It should be noted that these effects were obtained in a context in which both training regimens respected a progressive RT approach. This is relevant because objective load adjustments, based on the individual progression on a session-by-session basis, tend to promote a consistent increase in the physiological demands during workouts (overload principle).^6^ In addition, they also occurred in a context of high training frequency (5 sessions per week), totaling 20 workouts at the completion of each intervention. According to the conclusions of a recent systematic review, the effects of LLBFR-RT on type I fiber CSA appear to be more pronounced with very frequent training sessions (>5 days per week).^45^ Moreover, there is a required minimum of 14 to 21 training sessions to reliably detect myofiber hypertrophy.^10^ The novelty of our findings is that, even in a context of high-training frequency up to a total of 20 exercise sessions, LLBFR-RT did not potentiate the hypertrophy of the soleus beyond that seen with HL-RT.

The lateral gastrocnemius, a muscle with a mixed composition of slow- and fast-twitch fibers, manifested hypertrophy only in response to HL-RT, while hypertrophy was not observed in the medial gastrocnemius after either training regimen. Compared with the other calf muscles, the contractile properties of the lateral gastrocnemius are compatible with faster twitches.^48^ Conceivably, this may explain why the CSA of lateral gastrocnemius exhibited greater sensitivity to HL-RT. Consistent with these findings, past research has shown that in untrained persons the CSA of type II muscle fibers increases with HL-RT, but not with LLBFR-RT.^43^ The differential adaptation of the medial versus the lateral gastrocnemius to HL-RT is also well supported by the available literature. While the hypertrophy of the medial gastrocnemius displays a considerable level of insensitivity to variations in set volume, this is not the case for the lateral gastrocnemius.^23^ One likely explanation for this effect resides on the fact that the lateral gastrocnemius remains relatively inactive during standing and gait compared with the medial gastrocnemius,^11,21^ raising the possibility that it has a greater potential training capacity due to underuse. Even though all participants included in the present study performed the plantar flexion exercise using a variation that is known to be ideal for the proportional improvement on both heads of the gastrocnemius (i.e., foot pointing forwards),^40^ all repetitions were completed with full range of motion, and this is not optimal for the hypertrophy of the medial gastrocnemius, which is known to respond best to partial range of motion repetitions completed at longer muscle lengths.^22^ Thus, it can be concluded that the attenuated hypertrophic response of the medial gastrocnemius to both interventions most likely resulted from of its lower margin of trainability at baseline, together with its suboptimal stimulation in a context involving full-range of motion plantar-flexion exercise.

### Muscle Strength

Both training regimens resulted in gains in dynamic and isometric muscle strength, and the magnitude of gains was not different between them. Thus, when prescribed at high frequency (5 days per week), LLBFR-RT might serve as an alternative to HL-RT to improve muscle strength during the course of a 4-week mesocycle. Based on a recent systematic review, the beneficial impact of LLBFR-RT on muscle strength tends to be close or sometimes similar to that resulting from HL-RT.^5^ There are ≥3 factors that may explain the lack of difference in strength gains between LLBFR-RT and HL-RT observed in the present study. First, to achieve muscle strength gains comparable with HL-RT, untrained people are required to complete >18 training sessions with BFR,^5^ and this was the case. Second, to elicit strength gains as HL-RT, BFR-RT has to apply individualized cuff pressure during exercise.^4^ Again, this was strictly respected in our study because all BFR training sessions were completed at 60% of arterial occlusion pressure taken at the beginning of each session. Third, there is compelling evidence that performing multiple strength assessments (i.e., 1RM) over the course of the study could affect the strength gains from the training regimen.^47^ The participants assigned to each training regimen were requested to complete frequent 1RM testing sessions (as detailed in Methods). This was done to enable the progressive adjustment of training load over time.

It is interesting to note that both interventions resulted in similar plantar-flexion strength gains, despite their differential effect on the hypertrophy of the triceps surae muscle. The HL-RT regimen resulted in hypertrophy of the soleus and the lateral gastrocnemius, whereas LLBFR-RT resulted in hypertrophy of soleus. There are possible explanations for the observed dissociation between change in muscle size and strength. It is generally thought that the increase in muscle size is important mechanistically for the increase in muscle function. However, there is no experimental evidence that support any paradigm in which muscle hypertrophy is a mechanism for increasing strength.^29^ Alternatively, there are several adaptations in the nervous system that may provide a partial explanation for strength gains after 4 weeks of RT, including changes in the primary motor cortex, spinal cord, and/or alterations in the motor neuron.^1,20,26^ The muscle fiber itself may also undergo changes that can increase strength independently of the change in muscle size (e.g., change in the composition of the myosin motors, modification in patterns of calcium release, and changes in the major components involved in the excitation-contraction coupling).^29^

### Limitations

This study is not without limitations. For instance, a nonexercise control group was not included. Without this, it cannot be confirmed that the outcome measures displaying a time effect increased because of the interventions per se. However, all analyzed variables are known to increase with RT and the magnitude of their change was consistently larger than the between-day variability obtained with a different group of untrained participants. In addition, because of their underrepresentation in BFR literature,^8^ women were included in this study. However, we did not control for the effects of the menstrual cycle on muscular strength or hypertrophy. While the menstrual cycle has been shown to have a trivial effect on force production characteristics,^2^ its impact on muscle hypertrophy may not be negligible because of the anabolic effect of estrogen.^25^ In addition, the small number of men and women in both groups did not allow us to compute sex comparisons due to limited statistical power. The length of both training regimens was limited to 4 weeks, which can be viewed as a short timeframe in terms of neuromuscular adaptation. Therefore, based on the present data, it is not possible to determine whether the adaptive responses to both modalities of RT might diverge or converge even further over a larger time frame. Finally, not all exercise sets were performed to failure during the course of training. The last set of all sessions was invariably completed to failure (in both training regimens), but the previous sets were terminated whenever each participant reached the prescribed number of repetitions or failure (whichever occurred first). Therefore, we cannot be certain that these findings still hold true in circumstances compatible with volitional failure throughout all exercise sets.

## Conclusion

The present data indicate both HL-RT and LLBFR-RT result in similar gains in size of the soleus. In contrast, neither intervention results in an increase in the size of the medial gastrocnemius and the 2 training programs differ only in their effect on the size of the lateral gastrocnemius. Despite these nuances, LLBFR-RT promotes gains in dynamic and isometric muscle strength that do not differ from those resulting from HL conditions.

### Practical Conclusion

High-frequency plantar-flexion exercise with LLBFR-RT (5 days per week) is well suited to practitioners aiming at partitioning size gains of the triceps surae with emphasis on the adaptation of the soleus. High-frequency plantar-flexion exercise with HL-RT (5 days per week) elicits size gains of the triceps surae with emphasis on the adaptation of both the soleus and the lateral gastrocnemius. The more regional nature of hypertrophy of the triceps surae with LLBFR-RT does not exert any negative impact on dynamic or isometric strength gains.
